# Bovine Lungworm: Prevalence and Associated Risk Factors in Haramaya Town, Ethiopia

**DOI:** 10.1155/japr/2776754

**Published:** 2026-01-17

**Authors:** Tsedalu Yirsa, Mohamed Galgalo

**Affiliations:** ^1^ Department of Veterinary Medicine, College of Agriculture, Woldia University, Woldia, Ethiopia, wldu.edu.et

**Keywords:** Baermann technique, cattle, lung worm, prevalence, risk factors

## Abstract

**Background:**

Lungworm infection, caused by a nematode parasite, leads to bronchitis or pneumonia, high‐mortality rates in cattle, and significant economic losses.

**Objectives:**

This study is aimed at assessing the prevalence of bovine lungworm and identify potential risk factors in Haramaya town, Ethiopia.

**Methods:**

A cross‐sectional study design was employed from December 2023 to April 2024, with animals selected using a simple random sampling method. Prevalence and associated risk factors were analyzed using chi‐square and logistic regression tests in Stata Version 17.

**Results:**

A total of 384 cattle fecal samples were collected and analyzed in the Haramaya veterinary parasitology laboratory for larvae identification. Of these samples, 16 (4.2%) tested positive for lungworm infection. Logistic regression analysis showed a statistically significant association between the disease and factors such as age, body condition, and management systems (*p* ≤ 0.05). The prevalence of lungworm infection was significantly higher in cattle with poor body condition (8.7%) than those in good condition (3.6% and 1.4%). Cattle with poor body condition were 0.6 times more likely (*OR* = 0.6, *C* *I* = 0.308–1.16) to be infected than those with good body condition. Cattle in extensive management systems had 4.26 times higher odds (*OR* = 4.26, *C* *I* = 1.16–15.62) of being infected compared with those in intensive management systems. Furthermore, young cattle were 0.23 times more likely (*OR* = 0.23, *C* *I* = 0.078–0.7) to be infected than adults.

**Conclusions:**

The relatively low prevalence of bovine lungworm in this region carries notable economic consequences. To mitigate these impacts, preventive measures such as vaccination and deworming should be implemented.

## 1. Introduction

Ethiopia hosts the largest livestock population in Africa, comprising approximately 70 million cattle, 42 million sheep, 52 million goats, 8 million camels, and 56 million chickens [1]. Consequently, the country′s average annual growth rate in the gross agricultural production value is 4.5%, which exceeds the African continental average of 2.2% [2]. This national herd is vital to the livelihoods of over 11.3 million rural households, including 27%–35% of highland livestock keepers and numerous lowland herders living below the poverty line [3]. Livestock provide essential resources such as meat, fat, milk, dairy products, eggs, and fibers like wool and cashmere, while also offering services including transportation, draft power, and fertilizer production, particularly in developing countries [4]. However, economic gains remain limited due to low livestock productivity caused by parasitic infections and poor husbandry practices [5]. Across 40 studies, fascioliasis‐related liver condemnation in cattle accounted for an estimated annual loss of 40,833,983 ETB (USD 6,417,848) [6], whereas a localized assessment of this parasite at the Wolaita Sodo municipal abattoir reported an additional annual loss of 1,505,856 ETB (≈ USD 43,024) [7]. Lungworms are widespread parasites of ruminants worldwide, causing respiratory conditions such as bronchitis and pneumonia [8]. *Dictyocaulus viviparus* (*D. viviparus*), a nematode that lives in the lungs and is a member of the family Dictyocaulidae within the order *Strongylida*, is the predominant lungworm species in cattle [9, 10]. The genus *Dictyocaulus* contains species like *D. viviparus*, which infects cattle and buffalo, and *Dictyocaulus filaria*, which mainly affects sheep and goats. The Dictyocaulidae family is a member of the superfamily *Trichostrongyloidea* [11].

The epidemiology of lungworm infections is intricate, and outbreaks are often difficult to predict [12]. Conditions such as warm summers combined with above‐average rainfall or brief periods of intense rainfall substantially increase infection rates [13]. The primary route of lungworm transmission is pasture contamination from infected animals [14]. Although lungworms are found worldwide, they are particularly common in temperate regions and in tropical and subtropical highlands, including Ethiopia [15]. The prevalence of lungworm infections in ruminants is influenced by multiple factors, including local climate, the presence of *Pilobolus* fungi, and other favorable environmental conditions [16]. Larval stages can enter a state of delayed development (hypobiosis) when exposed to cold before ingestion, potentially remaining in the host′s lungs for up to 5 months [17]. This hypobiosis is critical for the parasite′s year‐round survival in temperate regions [18]. Filth flies—including coprophagic species that feed on animal and human feces, and saprophagous species that consume waste, bedding, and decaying organic matter—play roles in feeding, oviposition, and reproduction [19]. As a result, various adult fly species, such as houseflies, act as natural carriers for over 100 pathogenic agents, including viruses, fungi, bacteria, and parasites worldwide [20].

*D. viviparus* exhibits a direct life cycle. Adult females deposit eggs in the bronchi, which are then coughed up and swallowed along with mucus. The eggs hatch into first‐stage (L1) larvae as they pass through the gastrointestinal tract and are subsequently excreted in the feces. Once on pasture, the larvae molt into second‐stage (L2) and then develop into the infectious third‐stage (L3) larvae. Grazing animals become infected upon ingesting these L3 larvae [21]. Clinically, lungworm infection ranges from mild coughing with slightly increased respiratory rates to severe, chronic coughing, known as verminous pneumonia. Key signs of associated bronchopneumonia include poor body condition, dyspnea, nasal discharge, weight loss, fever, and sometimes mortality [22]. Diagnosis is commonly confirmed using the Baermann technique, which detects L1 larvae in fecal samples. Control measures involve strategic deworming: treating all animals at the end of the rainy season to reduce parasite loads during grazing and before the rainy season at the end of the dry season to minimize pasture contamination [23]. Field studies in and around Bekoji town have documented reduced efficacy and suspected resistance to commonly used anthelmintics such as albendazole (and other benzimidazoles), levamisole (tetramisole), and ivermectin in naturally infected sheep [24, 25].

Although numerous studies from different regions of Ethiopia have documented the prevalence and risk factors of bovine lungworm and its effects on cattle health and productivity [9, 10, 15, 26, 27], available information on the overall distribution of the disease across the country is still limited. Reported prevalence varies widely depending on ecological conditions, management systems, and seasonal influences [9, 26]. However, no study has specifically examined bovine lungworm infection in Haramaya town and nearby areas. Local observations indicate that cattle in this region experience a respiratory condition suspected to be associated with lungworm infection, with patterns differing from those reported elsewhere. The absence of localized data restricts the development of targeted prevention and control strategies. Therefore, this study is aimed at filling this gap by assessing the prevalence of bovine lungworm and identifying related risk factors in Haramaya town and its surroundings. The findings will improve cattle health and productivity, inform better deworming and pasture management, and deepen understanding of lungworm epidemiology in Eastern Ethiopia.

## 2. Materials and Methods

### 2.1. Description of Study Area

**Figure 1:**
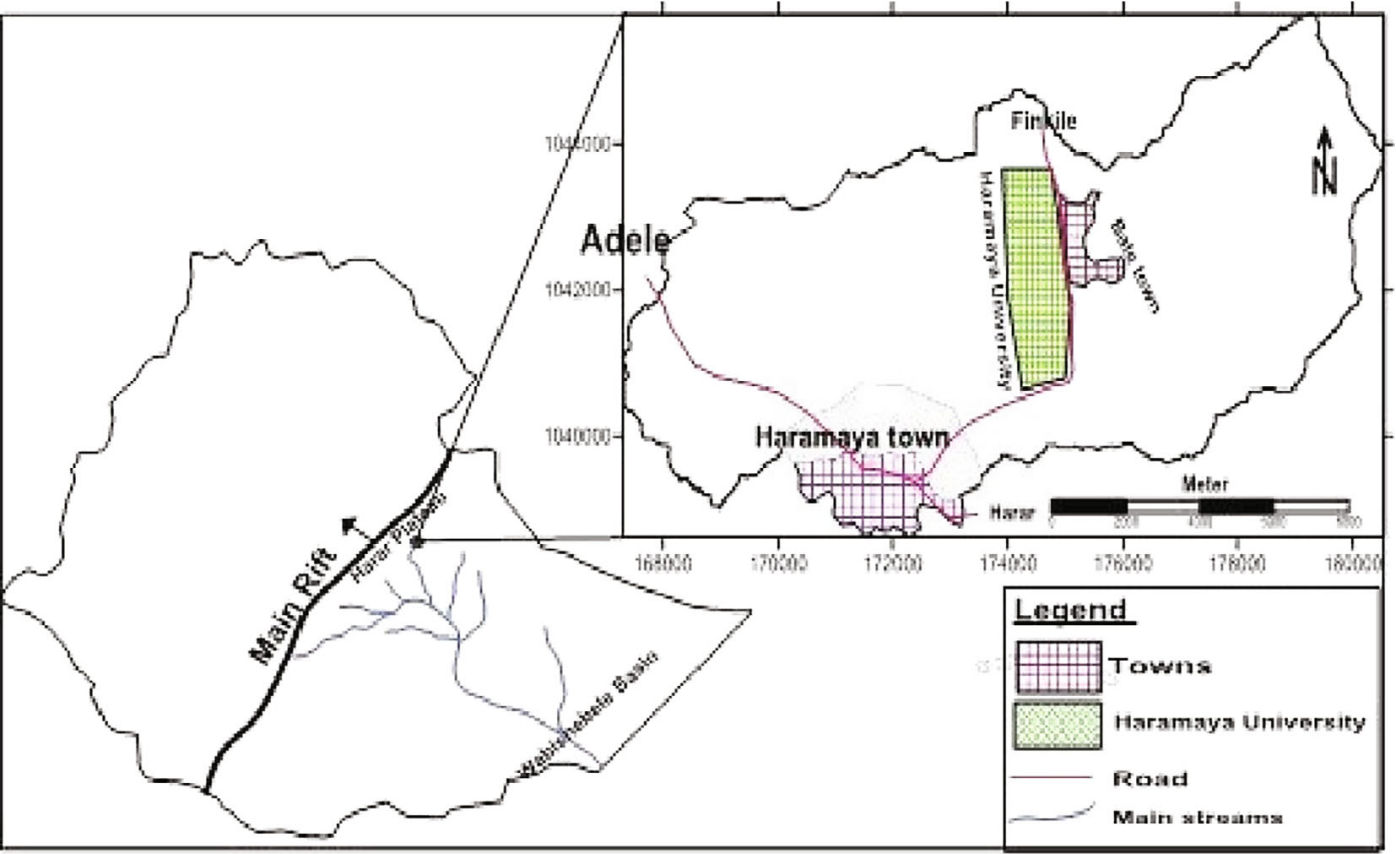
Map of the study area. Source: Google Map 2024.

This study was conducted in Haramaya City, Eastern Hararghe Zone, Oromia Region, Eastern Ethiopia, from December 18, 2023, to April 23, 2024. Haramaya is situated 14 km west of Harar and 508 km east of Addis Ababa, at 9°24 ^′^ N latitude and 42°01 ^′^ E longitude. The town lies at an elevation of approximately 1950 m above sea level, within a district altitude range of 1600–2100 m (Figure 1). The area experiences a bimodal rainfall pattern, with short rains from February to May, long rains from June to September, and a dry season from October to February. Average annual rainfall is 492 mm, ranging from 118 to 866 mm, whereas mean maximum and minimum temperatures are 17°C and 9°C, respectively. The estimated livestock population includes approximately 64,510 cattle, 18,930 sheep, 28,359 goats, 15,277 donkeys, 530 camels, and 65,723 poultry, with animals primarily managed under a mixed production system [28].

### 2.2. Study Design

A cross‐sectional study was conducted from December 2023 to April 23, 2024, to determine the prevalence of lungworm infection and assess associated risk factors in cattle in Haramaya town and its surrounding areas.

### 2.3. Study Populations

This study targeted cattle from Haramaya town and its surrounding areas, representing both extensive and intensive management systems. Cattle from six selected kebeles were included to obtain representative data for the entire region. The study sites were chosen based on criteria such as cattle population density, accessibility, and the availability of veterinary and statistical records, ensuring a comprehensive assessment of bovine lungworm prevalence. Sampling frames were established using complete cattle lists obtained from local statistical authorities and veterinary records. Key factors considered in determining the prevalence of lungworm infection included sex, age, body condition, and management system [9, 10, 26, 27]. Both male and female cattle were included in the study. Age was estimated using information provided by the owners and by examining dentition patterns, following the guidelines outlined by Tulu and Lelisa [29]. Thus, the age of cattle was classified into two age groups like as young (< 3 years) and adults (≥ 3 years). Additionally, the cattle′s body condition was assessed according to Nicholson and Butterworth′s criteria [30] as scored as *poor* (1–2; emaciated with prominent bones and minimal fat), *medium* (3; moderate fat cover and adequate condition), or *good* (4–5; well‐conditioned with abundant fat cover and a rounded appearance).

The cattle management strategies were categorized into two systems: extensive grazing, where animals spend most of the day freely grazing on open pasture, and intensive management, in which cattle are predominantly housed in barns or enclosures and fed using a cut‐and‐carry feeding system [10]. Cattle were divided into two breed groups for this study: crossbreeds and local (Zebu). Local Zebu cattle are native to Ethiopia and have adapted well to the local environment. They can survive on poor‐quality feed and are resistant to common diseases and heat. In contrast, crossbreeds are produced by mating local Zebu cattle with exotic breeds, like Jersey or Holstein‐Friesian, in order to combine the hardiness of the local breeds with the higher productivity traits of the exotic breeds, such as improved carcass quality, increased milk yield, and faster growth rates. Comparing the susceptibility of various genetic backgrounds to diseases, such as lungworm infections, is made possible by this classification [31].

### 2.4. Sample Size Determinations and Sampling Methods

The sample size for this study was calculated using the formula by Thrusfield et al. [32] assuming a 95% confidence level, 5% desired precision, and an expected lungworm prevalence of 50% in cattle.

n=1.962Pexp 1−Pexpd2

Where *P* *e* *x* *p* = expected prevalence; *d* = absolute precision; *n* = sample size. Thus, the estimated sample size would be 384 animals to determine the prevalence of this disease. A simple random sampling method, using a lottery technique, was applied to select study animals in herds with more than five cattle. Data recorded for each animal included age, sex, breed, body condition, and management system, reflecting biological characteristics and risk factors previously reported to influence lungworm occurrence [8, 9, 26, 27]. The study area comprised three woredas: Adele, Haramaya, and Awaday, each containing four kebeles. Two kebeles from each woreda were selected randomly: Tare and Golo (Adele), Mude and Bate (Haramaya), and Maya and Qore (Awaday).

### 2.5. Sample Collection and Laboratory Technique

Approximately 25 g of fresh fecal material was collected directly from the rectum of each animal using disposable gloves and placed into sterile, individually labeled stool containers. Each container carried a unique identification code corresponding to the animal′s herd, sex, age, body condition score, management system, and sampling date. The samples were stored in a cooler at 4°C and transported to the Veterinary Parasitology Laboratory, School of Veterinary Medicine, Haramaya University. A strict chain of custody protocol was maintained to ensure sample integrity and complete traceability during collection, transport, and handling. All samples were processed within 24 h of collection to prevent larval degradation or the development of hatching artifacts [33].

The modified Baermann technique was employed to recover L1 lungworm larvae from fecal samples following standard parasitological protocols [10]. Approximately 25 g of fresh feces was placed in a double layer of clean gauze and tied securely to form a pouch. This fecal pouch was then suspended in a glass beaker filled with approximately 500 mL of lukewarm water (25°C–30°C), ensuring that the feces remained fully submerged without touching the bottom of the beaker. The setup was left undisturbed for 24 h, allowing active larvae to migrate out of the fecal material and move downward into the surrounding water. After the incubation period, the fecal pouch was removed and discarded. The water containing the larvae was carefully poured into a test tube and allowed to stand for at least 30 min to permit sedimentation of the larvae at the bottom. The supernatant was gently decanted, and the sediment containing the concentrated larvae was transferred to a Petri dish using a Pasteur pipette. This sediment was examined under a stereomicroscope at low magnification to detect larval presence. When larvae were observed, a drop of the sediment was placed on a microscope slide, covered with a cover slip, and examined under a compound microscope for detailed morphological identification. The *D. viviparus* L1 larvae are slender, 300–360 *μ*m long, with a smooth, rounded anterior end and a filiform esophagus occupying about one‐third of the body. The tail is blunt with a small dorsal spine, and the larvae are active, unsheathed, with granular intestinal cells. These features distinguish them from other lungworm larvae such as *Muellerius* spp. and *Protostrongylus* spp. [9, 10, 26].

### 2.6. Data Management and Analysis

The collected data were coded, entered into Microsoft Excel, and analyzed using Stata Version 17. Descriptive statistics, including frequencies, percentages, and cross‐tabulations, were used to summarize the data. Additionally, chi‐square (*χ* ^2^) tests, binary logistic regression, and multiple logistic regression analyses were conducted to evaluate the associations between disease prevalence and potential risk factors. A *p* value of < 0.05 was considered statistically significant.

## 3. Results

### 3.1. Coprological Prevalence of Lungworm

A total of 384 cattle fecal samples, including 230 females and 154 males, were examined microscopically. Lungworm larvae (*D. viviparus*) were detected in 16 samples, yielding an overall prevalence of 4.2%. All larvae were identified as *D. viviparus* based on characteristic L1 morphology, including a smooth, rounded anterior end and a blunt tail with a dorsal spine [34]. The prevalence of infection varied with management system, being 1.8% in intensively managed cattle and 5.9% in extensively grazed cattle. When stratified by age, body condition, sex, and breed, prevalence rates were as follows: young cattle, 7.1% versus 2.2% in adults; poor body condition, 8.7%, medium, 3.6%, and good, 1.4%; females, 4.6% compared with 3.6% in males; and local breeds, 5.1% versus 2.7% in crossbreeds (Table 1).

**Table 1:** Cross‐tabulation analysis of risk factors for lungworm in cattle in the study area.

**Risk factor**	**Categories**	**Total examined animals**	**No. of positive animals**	**Prevalence (%)**	**Chi-square *(*** **X** ^2^ **)**	**p**
Management	Intensive	167	3	1.8%	4.1	0.041
	Extensive	217	13	5.9%		

Age	Young	155	11	7.1%	5.6	0.018
	Adult	229	5	2.2%		

BCS	Poor	103	9	8.7%	8.2	0.017
	Medium	140	5	3.6%		
	Good	141	2	1.4%		

Sex	Male	166	6	3.6%	0.22	0.64
	Female	218	10	4.6%		

Breed	Local	235	12	5.1%	1.34	0.25
	Cross	149	4	2.7%		
Total		384	16	4.2%		

Logistic regression analysis revealed a statistically significant variation (*p* value ≤ 0.05) in lungworm infection prevalence with respect to age, management system, and body condition score of the cattle. In contrast, no significant differences were observed for sex and breed (*p* value > 0.05). Young cattle were 0.7 times (70%) less likely to be infected than adult animals (*C* *O* *R* = 0.292). Cattle managed under extensive grazing were approximately three times more likely to be infected compared with those under intensive systems (*C* *O* *R* = 3.48). Animals with good body condition (1.4% prevalence) were less likely to harbor lungworm infection compared with those with poor (8.7%) or medium (3.6%) body condition. Cattle in good body condition had a 44% reduction in the odds of infection (*C* *O* *R* = 0.56) relative to cattle with lower body condition scores as summarized in Table 2.

**Table 2:** Logistic regression analysis of associated risk factors of infected cattle at the study area.

**Risk factor**	**N**	**n**	**COR (95% CI);** **p**	**AOR(95% CI) and** *p*
Age				
Young	155	11	0.292 (0.1–0.9) 0.025	0.23 (0.078–0.7) 0.009
Adult	229	5		
Management				
Extensive	217	13	3.48 (1.07–12.43) 0.050	4.26 (1.16–15.62) 0.029
Intensive	167	3		
Body conditions				
Poor	103	9	0.56 (1.3–2.07) 0.050	0.6 (0.308–1.16) 0.126
Medium	140	5		
Good	141	2		
Breed				
Local	235	12	0.5 (0.16–1.6) 0.255	—
Cross	149	4		
Sex				
Male	166	6	1.28 (0.46–3.6) 0.637	—
Female	218	10		

Abbreviations: N, no. of total examined; n, no. of positive; COR, crude odds ratio; AOR, adjusted odds ratio; Cl, confidence interval.

## 4. Discussions

The overall prevalence of Bovine lungworm was 4.2% in the study area. This finding was comparable with 3.75% in Gondar [26], 4.7% in Mendi town [9] from different parts of Ethiopia, and 4.76% in Pakistan [35]. The similarity across these studies is likely due to the use of comparable diagnostic methods, such as fecal larval detection or the Baermann technique, as well as shared agro‐climatic conditions during the wet season. Conversely, this finding was relatively higher than 0.3% in Turkey [36] and 3.02% in Nigeria [37]. These findings may be linked to poor management practices, such as continuous grazing and irregular deworming, as well as the prolonged wet season that favors the survival and development of *Dictyocaulus* larvae on pasture, all of which likely increased lungworm infection in the study area. On the other hand, this finding was lower than the previous reports of 24.39% in Durame [10], 30.1% in Woreta town [27], 34.3% in Tiyo districts [15], and 36.52% in Wolaita zone [38] from various parts of Ethiopia, and else in the globe including 13.98% of Iran [36], 20.98% in Egypt [39], and 22.64% in Rezvanshahr city of Iran [40]. This variation in lungworm prevalence among studies may be explained by differences in sample size and study populations, variations in laboratory protocols such as the modified Baermann technique, and the timing of sample collection across different seasons [9, 13, 22, 26].

A binary logistic analysis test demonstrated a statistically significant correlation between the occurrence of lungworm infection and risk factors such as age category (*p* value = 0.025), management practices (*p* value = 0.050), and the body condition of the animals assessed (*p* value = 0.050). The prevalence of lungworm infection in adult cattle was 0.7 times lower (2.2%) compared with young cattle (7.1%) (95% COR: 0.292, 95% CI: 0.1–0.9). These findings align with results reported in previous studies [9, 10, 15, 27, 39, 40]. Nevertheless, previous global studies have shown no significant correlation between the occurrence of lungworm infection and the age of cattle [26, 37, 38]. These differences may be related to young cattle being more vulnerable due to immature immunity and increased exposure to infectious larvae, whereas adult cattle have lower lungworm prevalence because of acquired immunity from previous exposure, more developed respiratory and immune systems, selective grazing behavior, better nutrition, and better body condition [38]. Although repeated exposure over time enables older cattle to develop acquired immunity that limits infection and clinical severity, calves are particularly vulnerable to lungworm infection during their first grazing season because they lack prior immunity, making them unable to effectively combat infectious *Dictyocaulus* larvae [13]. Consequently, young animals experience the heaviest infections and highest prevalence. In contrast, adult animals develop a rapid and solid immunity after the first infection, leading to persistent exposure to the parasite at a lower rate, which results in a decrease in the infection rates over time [13].

Regarding the management system, cattle kept in the extensive (5.9%) were 3.48 times more exposed than intensive (1.8%) management system (*p* value < 0.05) for this infection. This finding was agreed with the previous study reports from Ethiopia [9, 15, 38]. Nevertheless, the findings of Mahmood et al. [35] and Wondmnew et al. [27] stated that the incidence of this parasite has no significant relationship with cattle management practices. These variations can be attributed to the grazing practices of cattle in extensive systems, which often roam freely on pastures, particularly in communal or moist areas where infective *Dictyocaulus* larvae accumulate. Their susceptibility is further increased by uncontrolled housing and feeding, infrequent or irregular deworming, and limited veterinary supervision [27]. In contrast, cattle in intensive systems are typically housed, fed clean or stored fodder, and receive routine veterinary care and anthelmintic treatment, all of which reduce exposure to infective larvae and lower lungworm prevalence [38].

Regarding body condition scores, good body conditions (1.4%) was 0.44 times less exposed to this infection than medium (3.6%), and poor (8.7%) body condition scores (*p* = 0.050). These findings were closely agreed with the previous finding of various regions of Ethiopia [9, 10, 15, 26, 27, 41]. Nevertheless, a previous finding of Tessema et al. [38] stated that the prevalence of this parasite has no significant relation with the body condition scores. The difference attributed to stronger immunity and better nutrition help prevent larval establishment and control parasite burden, making good body condition scores of cattle less vulnerable to lungworm infection. On the other hand, cattle in medium or poor body condition frequently have weaker immunity, may be stressed or malnourished, and their health may be further compromised by prior parasitic infections, creating a vicious cycle of increased vulnerability [9, 39]. Body condition scores in cattle reflect their overall health and nutritional status, which strongly influence immune competence. Due to malnutrition, starvation, or stress, cattle in poor physical condition frequently have compromised immune systems, which makes them less resistant to *Dictyocaulus* larvae infection. Concurrent infections with other parasites, like gastrointestinal helminths, also weaken immunity and result in immunosuppression [27].

A key limitation of this study is that all larvae were identified as *D. viviparus* solely based on morphological characteristics, which may overstate the certainty of species identification. Although established diagnostic criteria and other larval features were used to confirm the presence of *D. viviparus*, molecular confirmation was not performed due to the lack of necessary reagents and equipment. This limitation has been acknowledged in the discussion, and future studies are recommended to include molecular methods to improve specificity and accuracy of larval identification.

## 5. Conclusion and Recommendations

The prevalence of bovine lungworm in the study area was 4.2%. This relatively low incidence highlights increased exposure in young animals, extensive management systems, and cattle with poor body condition. To prevent a significant parasite burden during grazing and reduce pasture contamination, regular grazing management and planned deworming of the entire herd with broad‐spectrum anthelmintics should be implemented. Additionally, isolating the most vulnerable age groups during peak pasture contamination periods is recommended. Further research on the molecular identification of this parasite in cattle is also suggested.

**Nomenclature**
BCS: body conditions
CI: confidence of interval
CSA: Central Statistical Agency
OR: odd ratio
TLU: tropical livestock units

## Ethics Statement

All procedures and animal care adhered to the Federations of Animal Sciences Societies (FASS) [42] guidelines, and the Animal Welfare and Ethical Review Committee of the Department of Veterinary Medicine of Woldia University approved the study (Ref. No. DVM/23/250/2023). All appropriate precautions were taken to minimize the pain the animals experienced in this study. Notably, there were no known dangers or discomforts related to taking samples from the study animals.

## Consent

Oral consent was provided to the study participants

## Disclosure

The authors read and approved the manuscript.

## Conflicts of Interest

The authors declare no conflicts of interest.

## Author Contributions

Mohamed Galgalo: write original documents, conceptualizations of the study, methodology, validation, and data collection. Tsedalu Yirsa also performed the statistical analysis, used software, and performed supervision.

## Funding

No funding was received for this manuscript.

## Data Availability

The data are available on request from the corresponding author due to privacy or ethical restrictions.
